# The need for speed

**DOI:** 10.7554/eLife.32009

**Published:** 2017-10-31

**Authors:** Tom Pizzari

**Affiliations:** Edward Grey Institute, Department of ZoologyUniversity of OxfordOxfordUnited Kingdom

**Keywords:** *Oncorhynchus tshawytscha*, sperm competition, social status, ejaculate quality, sperm velocity, seminal fluid, Other

## Abstract

A change in social status can quickly lead to a change in the quality of the seminal fluid produced by a male Chinook salmon as he responds to increased reproductive competition from higher-status males.

**Related research article** Bartlett MJ, Steeves TE, Gemmell NJ, Rosengrave PC. 2017. Sperm competition risk drives rapid ejaculate adjustments mediated by seminal fluid. *eLife* **6**:e28811. doi: 10.7554/eLife.28811

Males compete fiercely with other males for access to reproductive partners (Darwin, 1871). However, females often mate with multiple males, which means that the ejaculates of the males have to compete with each other in a process known as sperm competition (Parker, 1970). Competition between males can also be modulated by social hierarchy: dominant males can monopolise access to females, reducing the risk of sperm competition, and making subordinate males much more likely to face sperm competition whenever they manage to mate. Theory predicts that males facing higher levels of sperm competition should preferentially invest in the production of competitive ejaculates, and there is substantial empirical support for this (Parker and Pizzari, 2010).

In certain species of fish, for example, some territorial males rely on physical size to attract females, while others 'steal' paternity from the territorial males by sneaking into their territories and releasing more competitive ejaculates (Neff and Svensson, 2013). In such situations the sneaker males can only hope to fertilise a female if their sperm outcompete the sperm of a territorial male: consequently, sneakers adopt a developmental strategy that invests preferentially in gamete cells rather than somatic cells. However, social hierarchies can change rapidly as males join or depart social groups, or as individual males become older, and this has an impact on sperm competition. Moreover, it often takes weeks to produce sperm (a process known as spermatogenesis), and in some invertebrate species males emerge as adults with a fixed batch of ready-made sperm. In such cases how can males respond to an increased risk of sperm competition caused by sudden changes in their social status?

The answer to this question may be found in the seminal fluid. This is the non-sperm component of an ejaculate and it comprises of a diverse set of molecules, including hundreds of proteins secreted by different parts of the male reproductive tract and accessory glands. Increasing evidence indicates that the molecules in the seminal fluid have a strong influence on the processes that follow ejaculation and insemination (Poiani, 2006; Perry et al., 2013). These processes include female ovulation, the female propensity to remate, and female sperm storage. The molecules in the seminal fluid also influence the ability of the sperm cells to fertilize eggs, but relatively little is known about the many mechanisms through which this happens. In internally fertilising species, in which females store sperm in specialised organs, some proteins in the seminal fluid can help the sperm to reach the female's sperm storage organs (Wolfner, 2007). It is also known that fast sperm are often more likely to fertilise an egg than slow sperm (Snook, 2005; Pizzari et al., 2008), and the seminal fluid is thought to influence sperm velocity.

A number of studies have suggested that sperm swimming velocity may be negatively correlated with male social status in some species (Froman et al., 2002; Rudolfsen et al., 2006), suggesting that subordinates compensate for their lower status by preferentially investing in the competitiveness of their ejaculates. Moreover, in some cases it appears that a change in social status can quickly lead to a change in ejaculate quality: in the Arctic charr, for example, a male who becomes dominant over a rival male starts to produce slower sperm four days after his change of social status (Rudolfsen et al., 2006).

The present author has seen similar behaviour in experiments with domestic fowl (Pizzari et al., 2007). Males were assembled in pairs, generating a dominant and a subordinate. New pairs were then formed by placing two dominant males with each other, and two subordinate males with each other: this forced one male in each new pair to change his status and created four categories: males who were dominant in both pairs; males who switched from dominant to subordinate; males who switched from subordinate to dominant; and males who were subordinate in both pairs. As in the case of the Arctic charr, these changes in the social hierarchy led to changes in sperm velocity. In both cases the changes in sperm velocity occurred on a time scale that is faster than the rate of spermatogenesis, which rules out the possibility that they might be due to the production of sperm cells with a different phenotype. However, it is possible that the changes in sperm velocity are caused by changes in the biochemical make-up of the seminal fluid.

Now, in eLife, Michael Bartlett of the University of Canterbury and colleagues – Tammy Steeves (Canterbury), Neil Gemmell and Patrice Rosengrave, both from the University of Otago – report the results of experiments on the Chinook salmon, an externally fertilising fish, that provide convincing evidence for the hypothesis that the seminal fluid mediates rapid changes in sperm velocity (Bartlett et al., 2017). Male Chinook salmon (*Oncorhynchus tshawytscha*) fight for social dominance, with the winner gaining privileged access to spawning opportunities, and the loser having to adopt a sneaker strategy. This species therefore presents an excellent opportunity to study changes in sperm quality induced by social status.

The study comprises of three experimental steps. In the first step, Bartlett et al. exposed individual males to a double social challenge (similar to what Pizzari et al., 2007 did with fowl). Males were stripped of their semen (milt) and sperm velocity was measured 48 hours following each social challenge: it was found that subordinate males produced ejaculates with higher sperm concentration and faster swimming sperm than dominant males. The differences in concentrations emerged 48 hours after the first social challenge. The differences in velocity, on the other hand, became pronounced only after the second social challenge. This effect was largely driven by a noticeable increase in sperm velocity in males who had switched from being dominant to being subordinate. This pattern is broadly consistent with the idea that males who lose out in social competition strategically compensate by investing preferentially in competitive ejaculates.

To investigate whether the observed changes in sperm velocity are due to seminal fluid, Bartlett et al. conducted a second set of experiments in which the sperm of a male were incubated *in vitro* in the seminal fluid of his rival or, as a control, his own seminal fluid. They found that the sperm of a dominant male swim faster when they are incubated in the seminal fluid of the subdominant rival, and that the sperm of a subdominant male swim faster when they are incubated in his own seminal fluid. This confirms that when a dominant male becomes subdominant, he responds quickly by increasing the quality of the seminal fluid in his ejaculate, which increases his chances of fertilizing the eggs of any female he manages to spawn with.

In a third set of experiments the eggs of a female were exposed *in vitro* to ejaculates of similar size from two rival males, and the numbers of alevins fathered by each male were counted. The number of alevins fathered by subordinate males was higher than the number fathered by dominant males by a small but significant amount. And again, the sperm of dominant males were more successful when they were in the seminal fluid of subdominant males, and the sperm of subdominant males were less successful when they were in the seminal fluid of dominant males. In a separate set of trials, eggs were exposed to the sperm of a single male mixed with either his own seminal fluid or the seminal fluid of the rival male. Again, higher sperm velocities led to increase reproductive success.

Collectively, these results represent the most convincing case so far that social status influences the fertilising efficiency of sperm via the seminal fluid, and they also open up a number of questions. First, why don’t males always produce high-quality seminal fluid to maximise sperm velocity in all ejaculates? Specifically, what prevents dominant males from doing this? It is likely that the cost of producing high-quality seminal fluid is so high that males are unable to achieve social dominance and produce high quality ejaculates at the same time. If so, this study identifies a mechanistic pathway through which genetic variance can be maintained in the face of intense sexual selection in polyandrous populations. When males compete both before and after mating or spawning, as happens in the Chinook salmon and many other species, they can preferentially invest in either pre- or post-copulatory competition. Therefore, rather there being directional selection for a single phenotype, this trade-off can foster disruptive selection and the evolution and maintenance of alternative mating tactics.

It would also be useful to know which specific molecules in the seminal fluid molecules underpin these effects. In principle, seminal fluid can modulate sperm motility by providing substrates for energy metabolism (such as glucose). However, it is also possible that proteins in the seminal fluid may be implicated in more complex effects: for example, they might modulate the bioavailability of certain compounds through enzymatic activity or buffer the damage caused by reactive oxygen species. Alternatively, some seminal fluid molecules may be delivered to sperm cells and contribute to their structural properties (Girouard et al., 2009; Corrigan et al., 2014) and, possibly, their motility as well. And once the molecules responsible have been identified, attention can turn to understanding how these molecules influence sperm competition.

A related question concerns the phenomenon of ejaculate exploitation, in which males take advantage of the ejaculates from other males who have already copulated with a given female in order to increase their chances of reproducing at a minimal cost (Sirot et al., 2011; Alonzo and Pizzari, 2010). In the grass goby (*Zosterisessor ophiocephalus*), for example, the sperm of sneaker males appear to be able to take advantage of seminal fluid from territorial males (Locatello et al., 2013). This suggests that the sperm of dominant Chinook salmon might be able to benefit from the superior seminal fluid of subordinate males. The timing of competing ejaculations and their position relative to the eggs may also have an influence on which males are successful. It is also possible that the biochemical complexity of seminal fluid might enable interactions between the sperm and the seminal fluid that discriminate between sperm that are in their own seminal fluid and those that are in the seminal fluid of a rival. Understanding sperm competition and related phenomena like ejaculate exploitation will keep researchers busy well into the future.
